# Evaluation of Oil Displacement by Polysaccharide Fermentation Broth of *Athelia rolfsii* Under Extreme Reservoir Conditions

**DOI:** 10.3390/molecules30132861

**Published:** 2025-07-04

**Authors:** Haowei Fu, Jianlong Xiu, Lixin Huang, Lina Yi, Yuandong Ma, Sicai Wang

**Affiliations:** 1 School of Engineering Science, University of Chinese Academy of Sciences, Beijing 100049, China; fuhaowei22@mails.ucas.ac.cn (H.F.); wangsicai22@mails.ucas.ac.cn (S.W.); 2 Institute of Porous Flow and Fluid Mechanics, Chinese Academy of Sciences, Langfang 065007, China; huanglixin69@petrochina.com.cn; 3 National Key Laboratory of Enhanced Oil and Gas Recovery, PetroChina Research Institute of Petroleum Exploration and Development, Beijing 100083, China; yilina69@petrochina.com.cn (L.Y.); mayuandong69@petrochina.com.cn (Y.M.)

**Keywords:** polymer flooding, viscoelasticity, extreme reservoir, enhanced oil recovery, microbial oil recovery

## Abstract

In the development of high-temperature and high-salinity oil fields, biopolymer scleroglucan flooding technology faces significant challenges. Traditional scleroglucan products exhibit poor injectability and high extraction costs. This study investigated the application potential of the original fermentation broth of exopolysaccharides (EPS) produced by microorganisms in a simulated high-temperature and high-salinity oil reservoir environment. The polysaccharide was identified as scleroglucan through IR and NMR analysis. Its stability and rheological properties were comprehensively evaluated under extreme conditions, including temperatures up to 150 °C, pH levels ranging from 1 to 13, and salinities up to 22 × 10^4^ mg/L. The results demonstrated that EPS maintained excellent viscosity and stability, particularly at 76.6 °C and 22 × 10^4^ mg/L salinity, where its viscosity remained above 80% for 35 days. This highlights its significant viscoelasticity and stability in high-temperature and high-salinity oil reservoirs. Additionally, this study, for the first time, examined the rheological properties of the original fermentation broth of scleroglucan, specifically assessing its injectability and enhanced oil recovery (EOR) performance in a simulated Middle Eastern high-temperature, high-salinity, medium-low permeability reservoir environment. The findings revealed an effective EOR exceeding 15%, confirming the feasibility of using the original fermentation broth as a biopolymer for enhancing oil recovery in extreme reservoir conditions. Based on these experimental results, it is concluded that the original fermentation broth of *Athelia rolfsii* exhibits superior performance under high-temperature and high-salinity conditions in medium–low permeability reservoirs, offering a promising strategy for future biopolymer flooding in oil field development.

## 1. Introduction

Polymer flooding is a highly efficient enhanced oil recovery (EOR) technology that has been successfully applied on a large commercial scale in various oil fields [1], including Daqing Oilfield in China, Brintnell Oilfield in Canada, Mangala Oilfield in India, Captain Oilfield in the British North Sea, and Vacuum Oilfield in the United States [2,3,4,5]. These fields primarily utilize locally hydrolyzed polyacrylamide as a polymer flooding agent due to its excellent viscosity-enhancing properties and relatively low cost. However, the harsh environmental conditions of high-temperature and high-salinity oil reservoirs impose stringent requirements on the oil recovery process. Traditional polymer flooding methods face significant challenges in such reservoirs due to the poor thermal and salt tolerance of polyacrylamide [6,7]. Consequently, the oil industry has gradually turned its attention to biopolymers, such as xanthan gum and guar gum, which have been implemented in some oil fields [8,9]. Despite these efforts, most biopolymers remain in the laboratory research stage, with many being eliminated due to cost and efficacy concerns [10,11].

The extracellular polysaccharide (EPS) produced by *Athelia rolfsii* exhibits excellent thermal and salt resistance properties. This polysaccharide, also known as scleroglucan or *Athelia rolfsii* polysaccharide [12], has a unique structural motif. Its repeating unit consists of four sugar residues, with every three residues featuring a beta-(1,6)-glycosidic bond branch on a neutral beta-(1,3)-glycosidic bond backbone [3,13]. As a non-ionic biopolymer, scleroglucan forms a stable rigid triple helix structure in aqueous solutions. 

The fermentation broth of scleroglucan has advantages such as being more environmentally friendly and lower in cost compared to the dry powder. Commercial scleroglucan is typically sold as a dry powder, which is commonly used by the oil industry to formulate oil displacement agents and drilling fluids, especially for high-temperature reservoir applications [14,15]. However, the application of scleroglucan in high-temperature and high-salinity reservoir environments faces several challenges. To date, no systematic study has been conducted on the reservoir adaptability of the original scleroglucan fermentation solution. Current research predominantly focuses on the gel properties, molecular structure, and fermentation production of scleroglucan. Moreover, most studies utilize commercially available scleroglucan dry powder, which significantly differs from the properties of the emulsion (fermentation solution) at the end of fermentation [16,17,18]. These differences extend beyond solubility, viscosity, and stability [19]. Therefore, it is particularly important to investigate the performance of scleroglucan emulsion under reservoir conditions.

The large-scale application of dry powder scleroglucan still faces several challenges, particularly regarding its high cost. Xia et al. reported that the price of commercial scleroglucan powder is approximately US $50/kg, which is significantly higher than that of other biopolymers such as xanthan gum (US $12/kg) and guar gum (US $2/kg) [20]. This highlights the cost-related challenges associated with the use of scleroglucan in enhanced oil recovery (EOR) applications. Another major drawback of scleroglucan is its poor filterability, which poses significant challenges for its field implementation in EOR processes. 

The filterability of scleroglucan is primarily limited by cell debris, polymer aggregates, and residual proteins produced during the fermentation process [21]. The complete dissolution of dry powder is a lengthy process, during which large particles often fail to dissolve and aggregate, failing to meet injection requirements. Stirring, filtration, heating, and other processing steps further increase operational costs. Seright’s study on the core transport properties of scleroglucan demonstrated that injecting a 900 ppm dextrose solution into a core with a permeability of 106 mD led to a steep increase in injection pressure and the formation of a colloidal layer at the inlet section [22]. This phenomenon underscores the need to consider multiple factors, such as core permeability, injection concentration, and sample treatment, when evaluating the performance of scleroglucan in core experiments. 

However, recent studies have shown promising results in improving scleroglucan’s injectability. Song’s core transport experiments indicated that a 1000 ppm scleroglucan solution can be successfully transported through medium-permeability cores (200–500 mD) without causing surface blockage [23]. Additionally, Xia et al. demonstrated that a 1000 ppm scleroglucan solution exhibited good injectability in cores with a permeability ranging from 120 to 1700 mD. These findings suggest that further optimization of scleroglucan formulations and processing conditions could enhance its applicability in high-temperature and high-salinity reservoirs [24].

There have been sufficient studies to demonstrate that salinity and temperature have minimal effects on the viscosity of scleroglucan. However, these studies typically focus only on apparent viscosity, with insufficient research on the viscosity, elasticity, and complex modulus of scleroglucan in variable reservoir environments. Additionally, the reservoir adaptability of scleroglucan is insufficient, especially under extreme reservoir conditions. The viscosity changes and rheological properties of scleroglucan need to be further evaluated and optimized. Currently, most studies concentrate on the gel properties, molecular structure, and fermentation production of scleroglucan [16,17,18]. The majority of these studies utilize commercially available scleroglucan dry powder, which has significantly different properties than the emulsion (fermentation liquid) at the end of fermentation. Therefore, it is particularly important to investigate the properties of the original scleroglucan fermentation liquid under reservoir conditions. The application of scleroglucan in the form of the fermentation solution eliminates the cumbersome and costly processing steps [19]. 

This study aimed to explore the application potential of exopolysaccharides produced by a strain of microorganisms in high-temperature and high-salinity oil reservoirs. First, the structure of the product was determined through preliminary purification and structural analysis. Subsequently, the scleroglucan fermentation solution, without any chemical treatment, was directly produced by laboratory fermentation. The viscoelasticity and stability of the original fermentation solution were analyzed. Finally, its behavior under actual reservoir conditions was evaluated in a Middle East oil field with high temperature, high salinity, and low permeability, thereby assessing the feasibility of using the original fermentation liquid of scleroglucan in extreme condition reservoirs.

## 2. Materials and Methods

### 2.1. Experimental Materials

#### 2.1.1. Polymer Production

*Athelia rolfsii* is stored in the National Key Laboratory of Enhanced Oil and Gas Recovery, Research Institute of Petroleum Exploration and Development, Beijing, China. The research object in this paper, the original fermentation broth of scleroglucan, was produced by this strain of microorganism.

#### 2.1.2. Culture Medium

Potato glucose AGAR (PDA) medium was prepared as follows: First, 200 g peeled and chopped potatoes were boiled, filtered to extract the liquid, mixed with 20 g glucose and 15 g agar, and water was added to bring it to 1000 mL. It was heated until the agar completely dissolved and its natural pH (5.6 ± 0.2) was confirmed. Then, it was aliquoted into culture containers and subjected to high-pressure steam sterilization. The seed medium (g/L) composition was as follows: glucose 30, yeast powder 3, potassium dihydrogen phosphate 2, potassium chloride 1, ferrous sulfate 0.5, magnesium sulfate 0.5, citric acid 1.0. It was aliquoted into culture containers and subjected to high-pressure steam sterilization. The fermentation medium (g/L) composition was as follows: glucose 50, sodium nitrate 3, potassium dihydrogen phosphate 2, potassium chloride 0.5, ferrous sulfate 0.25, magnesium sulfate 0.5, citric acid 1.0. It was aliquoted into culture containers and subjected to high-pressure steam sterilization [25,26]. The specific information of the chemical reagents is shown in Table 1.

#### 2.1.3. Instruments and Equipment

In this study, a variety of instruments and equipment were utilized to ensure the accuracy and reliability of our experimental results. The following table provides a detailed list of the major instruments and equipment employed, including their specifications and sources. The main instruments and equipment used in the study are shown in Table 2.

### 2.2. Quantitative and Structural Analysis of Polysaccharides from Athelia rolfsii

#### 2.2.1. Extracellular Polysaccharide Solution Was Obtained

The mycelium of *Athelia rolfsii* stored on a plate was inoculated into sterile seed medium as the seed solution. After the seed liquid was cultured for 4 days, the seed liquid was inoculated into medium at a 5% inoculation rate and incubated for 4 days to obtain the polymer fermentation solution. All culture conditions were carried out at 30 °C and the rotating speed of the shaking table was 200 rpm. The cells and insoluble substances were effectively removed from the fermentation solution. First, the fermented solution sample was treated, diluted with 2 times the volume of distilled water, heated and stirred, and the precipitated substances were separated in a centrifugal tube under a centrifugal force of 10,000× *g* for 15 min (30 °C). The supernatant was used as the solution for the follow-up study. The main components of the pellet are fungi and other insoluble impurities and polysaccharides that exist in the supernatant, and the clear liquid contains inorganic salts, residual sugars, other organic components, etc. [27,28]. All experimental data presented in this study were obtained from at least three independent replicates, with rheological parameters for each sample derived by averaging multiple measurements.

#### 2.2.2. Quantitative Method of Crude Polysaccharide

A certain amount of the supernatant obtained by centrifugation was taken and anhydrous ethanol equivalent to twice its volume was added to induce the precipitation of the biopolymeric products due to the polarity of the solvent, and stand overnight at 4 °C to ensure that the biopolymer products can fully precipitate. After standing, a high-speed centrifuge was used at a relative centrifugal force of 10,000× *g* for 15 min to collect the precipitated crude biopolymer. In order to remove possible residual impurities, the precipitate was rinsed with anhydrous ethanol to further purify the precipitated scleroglucan. Subsequently, the rinsed polysaccharide was placed in a drying oven at 90 °C to dry until the weight was constant to obtain a solid scleroglucan without water so as to accurately measure the polysaccharide content [28,29] in the solution.

#### 2.2.3. Analysis of Polysaccharide Composition and Structure

A certain amount of fermentation liquid was diluted three-fold with distilled water, stirred well, and centrifuged at 10,000× *g* for 20 min to remove the fungi, polysaccharides and other undissolved parts. Then, the supernatant was mixed with 2 times the volume of anhydrous ethanol and it was allowed to precipitate at 4 °C for more than 8 h. The precipitate was dissolved in distilled water and then 4 times the volume of anhydrous ethanol was added and it was allowed to precipitate at 4 °C for more than 8 h. The precipitate was removed and redissolved in distilled water to obtain the scleroglucan solid. Then, 5 g of the crude extracted sample was dissolved in 1 L distilled water. The polysaccharide was separated and purified [24] by ion exchange chromatography DEAE-Sephadex A25, and then freeze-dried to obtain the purified scleroglucan.

An element analyzer was used for quantitative analysis of carbon, hydrogen, nitrogen, sulfur and other elements in the purified substance. First, 1.0 g of sample was accurately weighed, placed in a tin dish, and sealed for testing. Instrument parameters: furnace temperature: 1030 °C; O mode; furnace temperature: 1060°C; elemental analysis. A sample of 0.1 g was dissolved in 100 mL water and the absorbance λmax (280 nm) was obtained with an ultraviolet spectrophotometer.

Weigh out 2 mg of the lyophilized scleroglucan sample and mixed with 200 mg potassium bromide. It was pressed into a tablet and examined in the range of 4000–400 cm^−1^ to determine the infrared absorption. During the nuclear magnetic test, an appropriate amount of sample was weighed and the biopolysaccharide was dissolved in DO_2_ solvent. The structure of the product was determined with a nuclear magnetic resonance spectrometer Bruker AVANCE III HD 400 M, and the polysaccharide configuration was further identified by C-13NMR at 400 MHz [30].

### 2.3. Evaluation of Rheological Properties of Polysaccharides Solution of Athelia rolfsii

#### 2.3.1. Study on Viscosity and Concentration Relationship of Exopolysaccharides

Using the quantitated polysaccharide fermentation solution, 1000 ppm, 2000 ppm, 3000 ppm, 4000 ppm, and 5000 ppm scleroglucan solutions were prepared by diluting the mother liquor. The viscosity of the biopolymer solutions at different concentrations was determined by using a Harker rotary rheometer (Frankfurt, Germany)at 90 °C and a shear rate of 7.34 s^−1^. The steady-state rheological properties of several concentrations of polymer solutions were tested according to the relationship between their apparent viscosity and shear rate. The shear rate range set in the experiment was 0.01–1000 s^−1^. The test temperature was held constant at 90 °C. A steady state rheological curve was fitted and analyzed by a power law function model.

#### 2.3.2. Evaluation of Temperature, pH and Salinity Tolerance of Exopolysaccharides

A batch of biopolymer solution with a concentration of 2500 mg/L biopolymer was prepared and tested at different temperatures, pH values and salinity. The viscosity of the biopolymer solution was measured at different temperatures (60–150 °C) and shear rates of 7.34 s^−1^ using a Harker rotary rheometer to explore the effect of temperature on the rheological properties of the biopolymer solution. The aliquoted solutions were adjusted to different pH values (1, 3, 5, 7, 9, 11, and 13) by adding an appropriate amount of 1 mol/L hydrochloric acid or 1 mol/L sodium hydroxide, and the viscosity of these solutions was determined by using a Harker rotary rheometer at 90 °C and a shear rate of 7.34 s^−1^. Due to the diversity of reservoir environments, this experiment evaluated the influence of pH on the viscosity of the biopolymer solution.

Brine solutions of different concentrations (0, 6, 40, 80, 150 and 220 g/L) were prepared to simulate the viscosity changes of the polymer solution under different salinity conditions. The composition of the brine is shown in Table 3. The mass ratio of sodium chloride:calcium chloride:magnesium chloride hexahydrate was 35:3:2. The viscosity was also measured at 90 °C and a shear rate of 7.34 s^−1^ by using a Harker rotary rheometer to study the influence of salinity on the stability and viscosity characteristics of the biopolymer solution.

#### 2.3.3. The Modulus Test of Polysaccharide Solution

Determining the Linear Viscoelastic Region (LVE) of a polymer is essential for accurately measuring its rheological properties. Within the LVE, a linear relationship exists between stress and strain, and the rheological behavior of the polymer solution remains stable over time. This stability minimizes measurement errors arising from material structural changes, such as relaxation or creep. In this region, the elastic and viscous moduli of the polymer solution can be approximated by the storage and loss moduli, respectively. For the experiments, the polymer solution was prepared at a concentration of 2500 mg/L, with a fixed oscillation frequency of 1 Hz, a temperature of 90 °C, and a shear stress range of 0.01–1 Pa.

Once the Linear Viscoelastic Region (LVR) of the polymer solution is established, it ensures that the applied stress does not induce non-linear behavior during oscillatory frequency sweep tests. The dynamic rheological properties of the solution at different frequencies were evaluated using frequency sweep tests. For these tests, the polymer solution was prepared at a concentration of 2500 mg/L, with a shear stress of 100 mPa applied at 90 °C to maintain the solution within the LVR. Frequency scanning was conducted over the range of 0.01–10.00 Hz to investigate the frequency dependence of the viscosity and elastic moduli.

Temperature scan tests were performed on the polymer solution under a fixed shear stress of 0.1 Pa to assess the changes in elastic and viscous moduli across a range of temperatures (30–90 °C). The polymer solution was prepared at a concentration of 2500 mg/L and adjusted to different salinities (0, 6, 80, and 220 g/L). The elastic and viscous moduli were measured at various temperatures under a fixed shear stress of 0.1 Pa and a constant oscillation frequency of 1 Hz.

#### 2.3.4. Long-Term Stability of Exopolysaccharides Under High Temperature and High Salinity Conditions

Polymer flooding typically involves a prolonged working cycle during which polysaccharides must maintain a high apparent viscosity under high-temperature and high-salinity conditions. Therefore, it is essential to evaluate the long-term aging characteristics of these polysaccharides. In this study, polymer solutions with a concentration of 2500 mg/L were prepared at different salinities (0, 6, 80, and 220 g/L). Each solution was transferred into a 50 mL Schlenk flask with a liquid volume of 25 mL. The flasks were evacuated to remove air and then purged with nitrogen to eliminate both the atmospheric oxygen and dissolved oxygen in the solution. The flasks were immediately sealed and placed in an incubator maintained at 76.6 °C for 35 days. Samples were withdrawn every five days to measure the changes in apparent viscosity.

### 2.4. Oil Displacement Effect Test

The primary parameters, brine, and oil samples of the selected Middle East reservoir were provided by the oilfield. The reservoir exhibited a permeability of 100–120 mD, a temperature of 76.6 °C, and a crude oil viscosity of 2.75 cP. The viscosities of the protoexopolysaccharide fermentation broths used for oil displacement were 10 cP and 20 cP, respectively.

The core flooding experiments were conducted as follows: After vacuum saturation, the core was initially saturated with brine overnight (as detailed in Table 3), followed by injection with dehydrated crude oil until no brine was displaced from the core. The core was then aged at 76.6 °C for 3 days. Subsequently, brine, biopolymer solution, and brine were injected successively at a constant rate of 0.5 mL/min, with the polymer injection volume set at 1 pore volume (PV). The second brine injection stage continued until the displacement pressure stabilized for each slug. The retardation factor (RF) and residual retardation factor (RRF) were calculated based on the pressure drop between different slugs. Here, RF is defined as the ratio of the pressure drop at the end of polymer flooding to that after primary water flooding, indicating the reduced mobility or increased flow resistance of the polymer solution in porous media. RRF represents the reduced permeability in the reservoir rock, determined by the ratio of the pressure at the end of water flooding after polymer injection to that after primary water flooding. Additionally, the recovery factor (%) and enhanced recovery factor (%) were calculated by Formula (1).

(1) $$RF=\frac{∆Pp}{∆Pwb}RRF=\frac{∆Pwa}{∆Pwb}$$

ΔPwb—pressure after a primary water drive, MPa;

ΔPp—pressure after biopolymer flooding, MPa;

ΔPwa—pressure after subsequent water flooding, MPa.

## 3. Results

### 3.1. The Composition and Structure of Polysaccharide Were Analyzed

Table 4 shows that the sample contains five elements: C, H, O, N and S, of which only a very small amount of N and S exist, 0.06% and 0.04%, respectively, indicating that the extracted material contains a very small amount of protein residue. In Table 5, the absorption peak of the ultraviolet spectrum of the solution shows that the absorbance at 280 nm is 0.0268, which also indicates that trace protein remains in the solution [31]. This result indicates that polysaccharide can be effectively purified by ethanol precipitation twice and then separated by a chromatographic column, meeting the conditions for subsequent structural detection and analysis.

**Figure 1:**
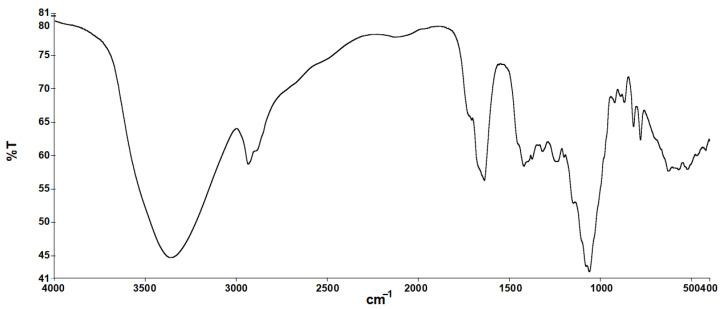
Infrared absorption spectrum of scleroglucan solution.

The infrared absorption spectrum of the sample is shown in Figure 1, revealing key features of its molecular structure. The most significant absorption peak shown in Table 6 is located at 3362.46 cm^−1^ and is attributed to the stretching vibration of the hydroxyl group (-OH) in the molecule. This wide peak indicates the presence of free hydroxyl groups in the scleroglucan fermentation solution, which is typical of a polysaccharide structure. Another significant absorption peak is located at 2936.82 cm^−1^, corresponding to the alkyl group (-CH_2_) contraction vibration, indicating the presence of an alkyl structure in the molecule. In the range of 1420.00~1200.03 cm^−1^, a series of absorption peaks were observed, which correlated with C–O stretching vibration in polysaccharides, reflecting the presence [32,33] of a sugar ring structure. In addition, the peaks at 1149.13 cm^−1^, 1079.06 cm^−1^ and 1060.86 cm^−1^ revealed the stretching vibrations of C–O–C and C–O–H bonds in the pyranose ring, further confirming that the polysaccharide in the fermentation solution conforms to the scleroglucan properties [34]. In particular, the absorption peak at 888.48 cm^−1^ is the characteristic vibration of a β-configuration polysaccharide, indicating that the polysaccharide in solution is connected [35] in the form of a β-glucoside bond. The results of these IR absorption spectra are consistent with the chemical structure of scleroglucan solution and the expected polysaccharide properties.

**Figure 2:**
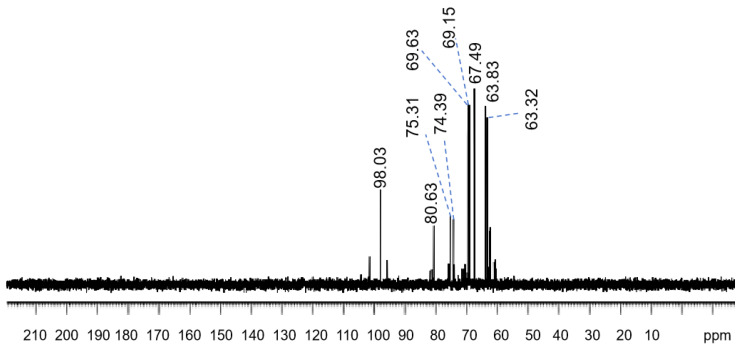
C-NMR spectra of scleroglucan.

**Figure 3:**
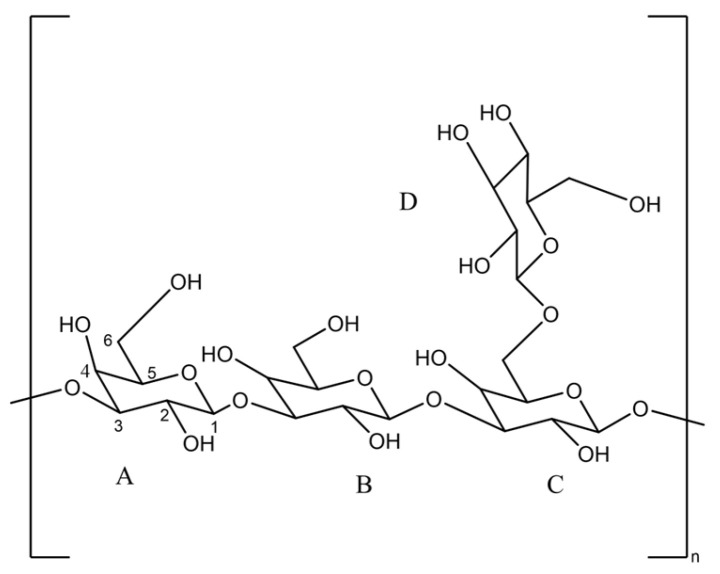
Scleroglucan repeated unit.

Further ^13^C-nuclear magnetic resonance (NMR) spectra of the polysaccharides solution provide information about its molecular structure. In the C-NMR spectra of the sample, there are mainly 9 groups of carbon signals, corresponding to 24 carbon atoms [32,36] in the structural fragment. The following is the attribution of each carbon atom. Each chemical shift value (δppm) in Figure 2 corresponds to the carbon atoms in the molecule under different chemical conditions. The signal at 98.03 ppm is due to the heterocephalic carbon atom attached to the hydroxyl group (C1-A,B,C,D), while the signal at 80.63 ppm and 75.31 ppm may be from the third carbon position in the sugar ring (C3-A,B,C, and C3-D). The peak at 74.39 ppm is attributed to C2-A, which is the carbon atom in the sugar ring that forms a glucosidic bond with two oxygen atoms. Peaks of 69.63 ppm and 69.15 ppm correspond to C2-B,C,D and C5-A,B,C,D, respectively, indicating that these carbon atoms are located in the second and fifth positions of the sugar ring. Peaks of 67.49 ppm are attributed to C4-A,B,C,D, the fourth carbon position in the sugar ring. Finally, peaks of 63.83 ppm and 63.32 ppm correspond to C6-C,D, and C6-A,B, respectively, carbon atoms that are typically attached [30,37] to hydroxyl groups. It can be inferred from these data (β-glucoside linked pyranose rings, detailed chemical shift values, and the linked hydroxyl groups on specific carbon atoms) that the polysaccharide structure matched [36,37] the scleroglucan structure. The fermentation liquid had the expected polysaccharide structure the type of carbon atom is given in Figure 3.

### 3.2. Steady-State Rheology Performance Evaluation Results

#### 3.2.1. Steady State Rheological Shear Test

**Figure 4:**
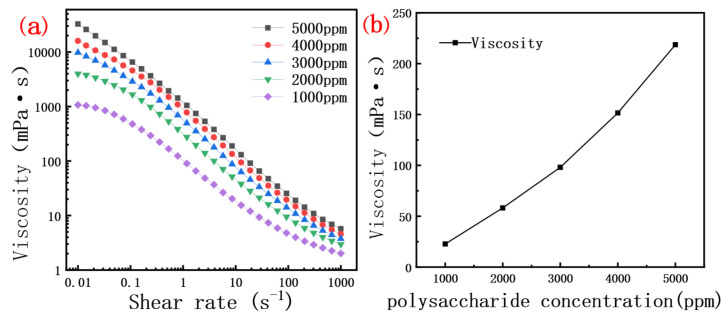
(**a**) Curve of viscosity of polysaccharide solution with different concentrations as a function of shear rate; (**b**) Curve of viscosity and concentration of polysaccharide solution (7.34 s^−1^ shear rate).

The viscosity of the crude polysaccharide solution was positively correlated with its concentration. The data in Figure 4a indicate that scleroglucan solutions of different concentrations exhibit a similar trend in viscosity under variable shear rates. The interaction and entanglement between molecules increased with the concentration of polysaccharide, Figure 4b shows the relationship between the concentration of dextran solution and viscosity. At low shear rates, the interactions and entanglements between glycosidic bonds molecules are minimal, allowing the molecular chains to move freely without significant internal structural changes [38]. This behavior is particularly evident at low concentrations, where the solution exhibits Newtonian fluid characteristics [39,40]. As the shear rate increases, the molecular chains of scleroglucan become more influenced by shear forces, leading to enhanced entanglement and interactions between chains. This increased entanglement complicates the internal structure of scleroglucan. Under external forces, the molecular chains cannot fully extend due to the higher molecular density in the system. Consequently, shear viscosity decreases with increasing shear rate, exhibiting pseudoplastic behavior. However, this behavior is more pronounced only at intermediate shear rates [41,42,43].

The power law model can be used to quantitatively describe the flow behavior [6,44] of polymer fluid within a certain shear rate range. The specific formula is as follows (2):

η = kÝ^n^ (2)

In the power-law model, k represents the consistency index and n denotes the dimensionless flow index, also known as the power-law coefficient. The fitted values of the power-law function, derived from the relationship between the viscosity of polysaccharide solutions at different concentrations and shear rate, are presented in Table 7. The shear rates selected for testing were from 1 to 1000 s^−1^, as the viscosity of the polymers is relatively stable within this range, and it covers a relatively wide range from low to high shear rates. When the value of R^2^ is close to 1, it indicates that the fitted power-law curve has a good correlation, and the results are reliable. All values of n are less than 1, indicating pseudoplastic behavior of the fluid, where the relationship between shear stress and shear rate is sub-linear. In other words, the apparent viscosity of the fluid decreases with increasing shear rate. Additionally, the n value decreases with increasing polysaccharide concentration, suggesting more pronounced pseudoplastic behavior and a stronger shear-thinning effect [45,46]. Conversely, the k value increases with increasing polysaccharide concentration. The thickening effect of the fluid is typically associated with increased internal structural resistance, such as enhanced entanglement between molecular chains or a more complex and tightly packed solution structure [45,46].

#### 3.2.2. Solution Stability at Different Temperatures, pH, and Salinity

**Figure 5:**
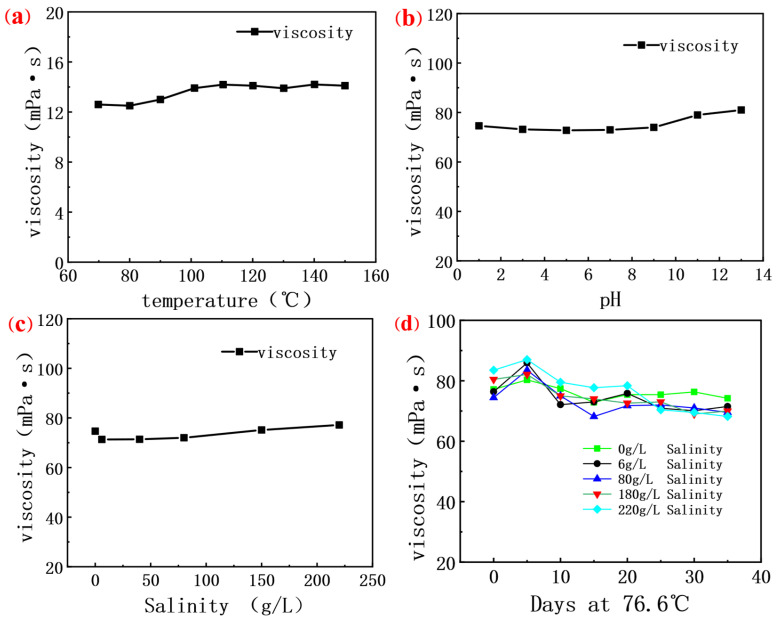
(**a**) Curve of viscosity of polysaccharide fermentation solution at different temperatures; (**b**) Curve of viscosity of polysaccharide fermentation solution at different salinities; (**c**) Curve of viscosity of polysaccharide fermentation solution under different pHs; (**d**) Curve of apparent viscosity of fermentation solution with aging time.

Figure 5a shows the viscosity of biopolymer solutions changes little when the temperature is increased from 60 °C to 150 °C. The stability of the viscosity is attributed to the fact that scleroglucan is a stable rigid triple helix structure in aqueous solution and this stable chemical bond structure gives it excellent thermal stability. Its molecular chains can also maintain a relatively stable configuration at high temperatures, thus tolerating high temperatures without a significant reduction in viscosity [13,47]. This shows that the biopolymer has good stability under high temperature conditions and is suitable for a reservoir environment above 120 °C. Looking further at the effect of pH on the viscosity of the solution in Figure 5b, it can be seen that the viscosity of the solution is higher than 70 mPa·s in the range of pH 1.0 to 13.0. This indicates that the biopolymer exhibits good stability under both strong acidic and strong alkaline conditions and can adapt to different pH environments.

In the case of increasing salinity, the viscosity of the solution is almost unaffected, as shown in Figure 5c where the viscosity of the salinity from 0–220 g/L is above 70 mPa·s, which is caused by the lack of functional groups in the molecular structure of scleroglucan that can strongly interact with salt ions, such as negatively charged carboxyl or phosphoric acid groups. This makes scleroglucan not susceptible to the interference of salt ions, and it can maintain its structure and function even in a high concentration salt solution, and it will not change the charge distribution [48,49,50] on the molecular chain, unlike other polyelectrolyte polymers that are affected by the pH value. This non-ionic property enables scleroglucan to maintain non-specific interactions between its molecules in a high salt environment, thus maintaining the viscosity and stability [51] of its solution.

#### 3.2.3. Long-Term Stability Studies

A 35-day aging experiment was conducted on four kinds of scleroglucan solutions with different salinities in a closed environment at 76.6 °C. The results shown in Figure 5d indicate that the viscosity of the solution without salt added remained stable, while the viscosity of the solution with salt ions added increased at the initial stage but then gradually decreased. This phenomenon can be attributed to the compression between salt ions and polymer molecular chains, which leads to the contraction of molecular chains, reducing the repulsion force between molecules and the spatial freedom. With the aging process, salt ions further destroy the entangled structure of the molecular chain, resulting in the weakening of the interaction forces between the molecular chains, entanglement fracture and settlement, thus reducing the overall viscosity [4,8] of the solution. In spite of this, the viscosity of these solutions can still maintain more than 80% of their initial value, indicating that although salt ions have a certain impact on the stability of the polymer solution, the viscosity of the solution has not been completely lost. In Table 8, the thermal resistance characteristics of some biological polymers are presented. Since general chemical polymers do not have good thermal resistance, they are not included as examples. It can be seen from the table that scleroglucan, which has a triple helix structure, exhibits excellent thermal resistance.

### 3.3. The Results Can Be Evaluated by Dynamic Rheology

#### 3.3.1. Linear Viscoelastic Region Test

Determining the Linear Viscoelastic Region (LVE) of a polymer is critical for measuring its rheological properties, where there is a linear relationship between the stress and strain of the polymer, meaning that the material behaves according to Hooke’s law, which states that the stress is proportional [6] to the strain. Beyond this region, the polymer will exhibit nonlinear behavior, which can lead to inaccurate measurements.

**Figure 6:**
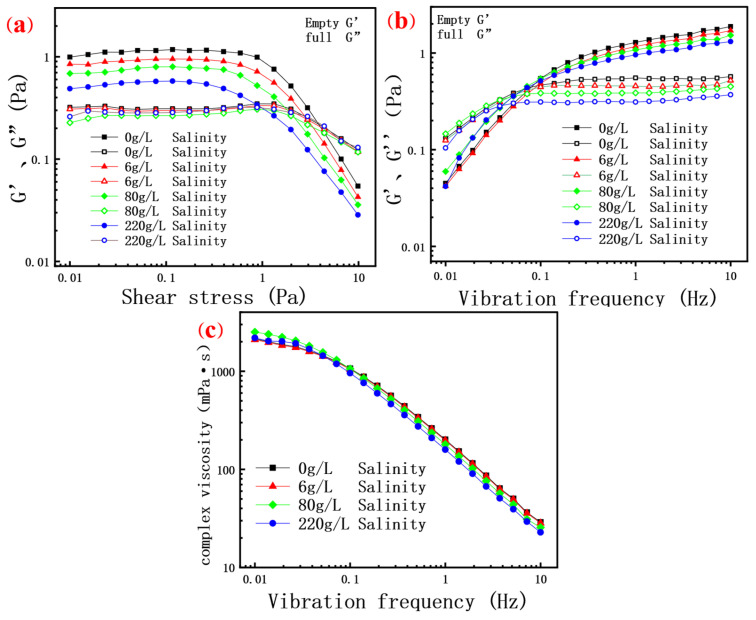
(**a**) The relationship between the elastic modulus G’ and the viscous modulus G” of the polymer solution at 0.01–10 Pa shear stress; (**b**) The relationship between the elastic modulus G’ and the viscous modulus G” of the polymer solution and the shear stress at 0.01–10 Hz oscillation frequency; (**c**) The relationship between complex viscosity and oscillating frequency of the polymer solution under 0.01–10 Hz shear stress.

In the linear viscoelastic region, the rheological behavior of a scleroglucan solution demonstrates its remarkable elastic properties. As shown in Figure 6a, the storage modulus (G’) of all scleroglucan solutions significantly exceeds the loss modulus (G”) in the low shear stress range tested, indicating that the elasticity of the solutions dominates when subjected to deformation [6,43]. Notably, at low shear stress, the elastic modulus (G’) decreases gradually with increasing salinity under the same shear stress, while the viscous modulus (G”) remains almost unaffected by salinity. This may be attributed to the compression of polymer molecules by added minerals, reducing the shrinkage ability of the molecules. Consequently, the molecular coils cannot undergo the process of shrinkage deformation and recovery under low or no salt conditions. As illustrated in the Figure 6a, in the high salinity curve (220 g/L), the elastic modulus (G’) decreases significantly at lower shear stress, further indicating that increased salinity restricts the shrinkage and elongation of polymer molecular chains. With increasing shear stress, G’ and G” fluctuate, but G’ consistently remains above G”, reinforcing the conclusion that a scleroglucan solution exhibits an elastic advantage under low shear stress.

However, when the shear stress exceeds 1 Pa, the rheological properties of a scleroglucan solution change significantly. Both the viscosity modulus and elastic modulus decrease, with the storage modulus (G’) decreasing more markedly, indicating that the viscoelastic properties of the solution shift toward viscosity under higher shear stress. This change may be related to the unentanglement and rearrangement of molecular chains under high stress, leading to alterations in the internal structure and intermolecular forces, thereby affecting the macroscopic rheological behavior [52]. In the shear stress range of 0.01–10 Pa, the viscosity modulus of the solution appears less affected by salinity. This is because the non-ionic biopolymer scleroglucan interacts with ions in the solution without charge. Even with added minerals, the friction between molecules under external force remains minimally affected, resulting in little change in the viscosity modulus.

#### 3.3.2. Oscillating Frequency Scanning Test

Under conditions of 90 °C and 0.1 Pa shear stress, the changes in elastic modulus, viscous modulus, and complex viscosity of the solution with oscillation frequency were investigated. As shown in Figure 6b, salinity appears to have a minimal influence on the viscosity modulus of the solution during oscillation frequency scanning tests. At low oscillation frequencies, the polymer solution’s molecular chains have sufficient time to respond to shear deformation and to rearrange and recover within each vibration period. This results in a relatively low elastic modulus, with the viscous modulus of all solutions exceeding the elastic modulus. When the oscillation frequency exceeds 0.1 Hz, the viscous modulus of scleroglucan remains essentially unchanged. Meanwhile, the elastic modulus (G’) is significantly greater than the viscous modulus (G”), indicating that the viscoelasticity of the solution is dominated by its elastic component. As the oscillation frequency increases, the elastic modulus rises while the viscous modulus remains nearly constant. The elastic modulus reflects the energy storage capacity of rigid molecules, which does not rely on molecular friction, whereas the viscous modulus is determined by internal molecular friction. However, at high frequencies, the molecules lack sufficient time to fully respond to the applied stress, resulting in limited strain and nearly unchanged frictional losses between molecules. Therefore, the viscous modulus does not change significantly with increasing frequency.

Additionally, as depicted in Figure 6c, the complex viscosity of the scleroglucan solution exhibits a strong correlation with oscillation frequency, with the complex viscosity decreasing significantly as the frequency increases. At low frequencies, polymer molecular chains have ample time to rearrange in response to shear forces, exhibiting pronounced viscosity and elasticity. However, as the frequency increases, the molecular chains cannot keep pace with the rapidly changing shear forces, leading to reduced energy dissipation (viscous contribution) and limited elastic contribution (due to rapid rearrangement of the chain segments).

#### 3.3.3. Effect of High Temperature and High Salt on Polymer Solution Modulus

**Figure 7:**
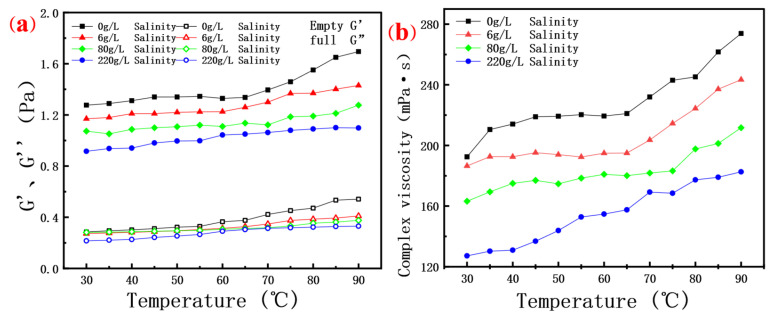
(**a**) The relationship between the elastic modulus G’ and viscosity modulus G” of the polymer solution and the temperature; (**b**) The relationship between the complex viscosity of the polymer solution and the temperature.

As depicted in Figure 7a,b, the elastic modulus, viscous modulus, and complex viscosity of several saline scleroglucan solutions exhibit variations with temperature. Specifically, the viscous modulus of these solutions only experiences a slight increase as the temperature rises, while the elastic modulus is more significantly affected by increasing salinity, a phenomenon that has been previously discussed and thus requires no further elaboration. The present discussion focuses on the impact of temperature on the modulus. Across the entire range of temperatures and salinities investigated, the elastic modulus consistently surpasses the viscous modulus. The rise in both moduli can be attributed to the enhanced motion of polymer molecular chains with increasing temperature. There exists a certain equivalent relationship between the increase in temperature and the increase in oscillation frequency in polymer solutions, which can be described by the Time–Temperature Superposition Principle (TTS) (References [45,53]). As the temperature rises, the rate of motion of the polymer molecular chains accelerates, leading to shorter relaxation and transition times. This corresponds to the polymer’s response at higher frequencies. Consequently, by increasing the temperature, the viscoelastic behavior of the polymer can be observed over a shorter period, an effect equivalent to that of increasing the oscillation frequency. In other words, elevated temperatures expedite the movement of molecular chains, enabling the observation of relaxation processes that would typically take longer at normal temperatures within a shorter timeframe. Moreover, the time–temperature equivalence principle is closely related to the WLF (Williams–Landel–Ferry) equation, which describes the relationship between temperature above the reference temperature and the time–scale transformation factor, further reinforcing the equivalence between temperature and frequency. This is consistent with the results of the oscillation frequency scan mentioned earlier, making it reasonable and understandable that an increase in oscillation frequency can be achieved by describing a similar increase in temperature. The complex viscosity also shows a significant increase with rising temperature. Since complex viscosity can be regarded as the total work done by external forces, during the temperature increase, both the energy W stored by the reversible deformation of the polymer and the frictional heat Q generated by irreversible deformation increase under the action of shear stress, resulting in a continuous upward trend in complex viscosity. A high complex viscosity can enhance the oil flow ratio, expand the swept volume, and improve the microscopic oil-washing efficiency during the oil-displacement process of the solution, thereby more effectively demonstrating the application potential of the solution under high-temperature conditions.

It should be highlighted that, in general, biopolymers such as xanthan gum, guar gum, welan gum, and fixed gum exhibit a softening behavior as the temperature increases, characterized by a decline in their elastic modulus, viscous modulus, and complex viscosity [6,20]. In contrast, the rigid triple-helix molecular structure of scleroglucan fermentation solution remains unaffected by temperature changes, which confers its distinct performance under elevated temperatures. This observation suggests that among the rheological studies conducted-namely, oscillation frequency scans, oscillation amplitude scans, and modulus measurements under varying temperatures-the temperature-dependent results are most closely linked to the rigid triple-helix structure. Previous studies have shown that the modulus of xanthan gum and fixed gum increases with rising oscillation frequency but exhibits poor thermal stability, as these polymers soften under elevated temperatures. This softening is attributed to the increased mobility of polymer chains at higher temperatures, which disrupts intermolecular interactions and reduces mechanical integrity.

### 3.4. Test Results of Oil Displacement Effect

The core displacement experiment was conducted at 76.6 °C and the concentration of inorganic salts was 220 g/L. A total of four core displacement experiments with similar permeability and core properties were carried out, and two parallel displacement experiments were carried out with 20 mPa·s and 10 mPa·s biopolymers, respectively, as shown in Table 9 with the specific parameters. In the primary water drive stage, most of the injection water channels of the high permeability layer are affected by gravity and the flow resistance of the middle and low permeability layer. Coupled with the high viscosity of crude oil, the water phase can easily break through in the oil reservoir, and the water content of the produced liquid increases rapidly in the initial stage of the primary water drive. There may be small differences in the primary water drive recovery rate of the four cores, but basically maintaining it at about 50% can ensure the parallelism of the experiment.

**Figure 8:**
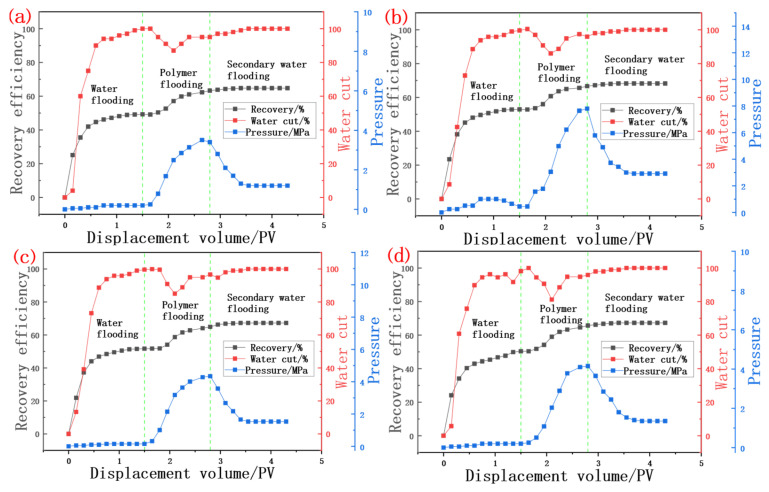
Curve of water content, recovery degree and pressure during polymer solution flooding. (**a**) Results of 10 cP polymer solution flooding experiment; (**b**) Results of 10 cP polymer solution flooding experiment; (**c**) Results of 20 cP polymer solution flooding experiment; (**d**) Results of 20 cP polymer solution flooding experiment.

As shown in Figure 8, the polymer can improve the sweep efficiency because of its macromolecular and viscous characteristics. In the polymer solution injection stage, due to the thickening ability of the scleroglucan solution, the pressure curve starts to rise from the pressure at the end of a primary water flood, and has a higher injection pressure in the late polymer injection stage. The late injection pressure of 20 mPa·s biopolymer flooding is about 4 Mpa, which is slightly higher than the 3 Mpa injection pressure of 10 mPa·s. In the core, there is a certain ability to block the hyperpermeability zone and thus reduce the permeability of the core. There is also a significant decrease in water content during polymer injection, and this decrease is maintained in the process of 0.5 PV polymer injection. In the subsequent water drive stage, due to the apparent viscosity of the injection agent the formation of water becomes smaller, the injection pressure begins to decrease, and the water cut rises to a higher level, gradually reaching more than 98%. The recovery rate of subsequent water drive is only slightly improved. As shown in Table 10, compared with 10 cP, the oil recovery rate of 20 cP biopolymer flooding increased from 15.35% to about 16.5% on average, and the resistance factor and residual resistance factor of the 20 mPa·s flooding experiment were also slightly higher. The residual resistance factor (RFF) and resistance factor (RF) were 23.9% and 19.0% higher on average, respectively. In summary, the polymer solution has a significant effect of improving oil recovery under high temperature and high salt environments.

## 4. Outlook

This study explored an original scleroglucan fermentation solution based on a simplified treatment process involving only dilution stirring and centrifugation steps. The main purpose of dilution stirring is to improve the utilization rate of polysaccharide and promote its dissolution. The dilution multiple adopted was three times. In industrial applications, although a larger dilution multiple is conducive to the dissolution of polysaccharide, it will also increase the cost of subsequent agitation and centrifugation steps. These factors will be further considered in subsequent studies. After a series of performance evaluations, the simple treatment of the original fermentation broth of polysaccharides from *Athelia rolfsii* fully meets the requirements of extreme reservoir applications. The fermentation broth not only has excellent temperature, salt and stability, but also has no blockage during injection in medium and low permeability reservoirs. Based on these results, the results of this study were [4,21,22,23,54] compared with those of other researchers.

**Figure 9:**
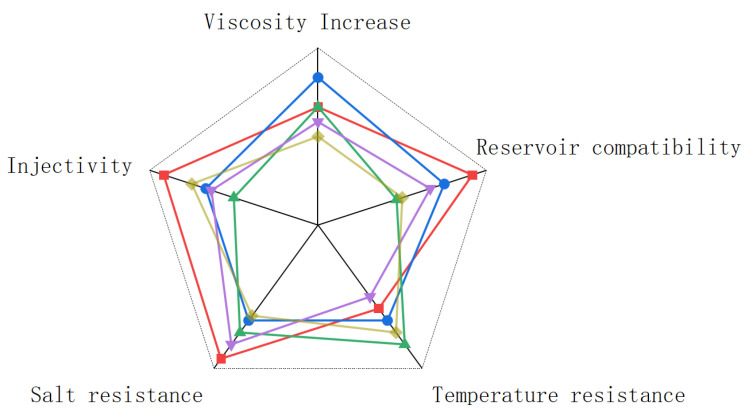
Comparison of several types of powder and colloidal scleroglucan in an oil field (all data have been standardized). The data for each parameter are represented in different colors: This work (red), Blue [6,23], Green [4,22], Purple [21], and Yellow [20,50].

As shown in Figure 9, a comprehensive comparison between the findings of this study and the studies of other authors shows that the simple treatment of the original fermentation liquid can fully meet the application requirements of high temperature and high salt oil reservoirs, especially in the aspects of salt tolerance, reservoir adaptability and injection where the original fermentation liquid shows excellent results. However, the performance of the original fermentation broth is not satisfactory from the perspective of increasing viscosity and temperature resistance, which may be due to the fact that the original fermentation broth has not been separated and purified, resulting in a variety of impurities in the fermentation broth that may have some impact on the stability of the fermentation broth. The stickiness of the original fermentation broth in this study may also be caused by overestimating the effective content of polysaccharides in the fermentation broth. After evaluating the application of the original fermentation broth in the reservoir, we should also consider the feasibility of this work. Since the original fermentation broth is a total nutrient solution, it is very easy to be contaminated with bacteria and cause the degradation of glycosidic bonds in the fermentation broth, resulting in loss. In future research, the fermentation site can be set at the oil production site to avoid this situation. The degradation of scleroglucan can also be reduced by anaerobic conditions and the addition of fungicides. Based on the research of predecessors, scleroglucan exhibits excellent rheological properties and can adapt to extreme reservoir conditions, mainly for the following reasons: First, it has superior thickening properties; second, it has rare viscosity stability; third, it retains good viscoelasticity under high temperature and high salinity; and finally, which is also very important, scleroglucan is a completely non-ionic polymer with a simple composition and poses no harm to the environment.

## 5. Conclusions

In this study, the performance of the original polysaccharide fermentation broth of *Athelia rolfsii* exopolysaccharides in a simulated high temperature and high salt reservoir was discussed for the first time, and the following conclusions were drawn by combining various research and evaluation methods:

(1) Structure and composition: elemental analysis and ultraviolet spectroscopy showed that the extracted polysaccharide contained a very small amount of protein residue, indicating that the purification process was effective. By NMR and IR analysis, it was confirmed that the polysaccharide had the structure of a pyranose ring connected by a β-glucoside bond, which showed the typical characteristics of scleroglucan. These results indicated that the original fermentation broth of the exopolysaccharides of *Athelia rolfsii* was structurally consistent with commercial scleroglucan.
(2) Stability: High temperature stability: At temperatures up to 150 °C, the viscosity of the scleroglucan solution showed little change, showing excellent thermal stability. pH stability: In the range of pH 1–13, the viscosity of the solution was higher than 70 mPa·s, indicating that it exhibits good stability under both strongly acidic and strongly alkaline conditions. This shows that scleroglucan is able to maintain its properties in a wide range of pH environments and adapt to reservoirs with different geological conditions. Salinity stability: At a salinity of up to 220 g/L, the viscosity of the solution remained above 70 mPa·s, showing excellent salt tolerance. Viscoelasticity: At low shear stress, the storage modulus G’ of scleroglucan solution was significantly higher than the loss modulus G”, indicating that it has significant elastic properties. Even under high salinity conditions, G’ is still higher than G”, indicating that its elastic advantage can be maintained at different salinities. This property enables the scleroglucan solution to better maintain its structure in the reservoir and improve the oil displacement efficiency. Long-term stability: At 76.6 °C and 220 g/L salinity, the viscosity of scleroglucan solution remained above 80% for 35 days, showing good long-term stability.
(3) Oil displacement effect: Scleroglucan solution can significantly increase oil recovery by more than 15% under conditions simulating high temperature and high salt reservoirs in the Middle East. When injected in medium and low permeability reservoirs, there is no core plugging phenomenon, indicating that it can effectively improve oil–water flow characteristics and improve sweep efficiency as a thickening agent. This result proves the application potential of scleroglucan fermentation broth under actual reservoir conditions, which can significantly improve oil field recovery.
(4) Excellent features of the original fermentation solution: Simplified processing, only requiring dilution stirring and centrifugation steps of the original fermentation solution, fully meeting the requirements of extreme reservoir applications without complex chemical treatment. This simplified process not only reduces the treatment cost, but also improves the treatment efficiency, making the original fermentation liquid more feasible in industrial applications. Under the condition of a medium and low permeability reservoir, there is no plugging phenomenon during the original fermentation fluid injection, which indicates that it has good injectivity. Although the viscosity of the original fermentation liquid is slightly lower than that of the purified scleroglucan, the processing is simple, the cost is lower, and the economy is higher. This characteristic makes the original fermentation liquid more cost advantageous in practical applications and it can reduce the cost of oilfield development.

In summary, because of its excellent performance in high temperature and high salt oil reservoirs, the original fermentation broth of polysaccharides from *Athelia rolfsii* has shown great potential as a microbial oil recovery technology. Future research should focus on further optimization of production processes, cost reduction, and application potential under different reservoir conditions so as to promote its wide application in oilfield development.

## Figures and Tables

**Table 1:** Chemical reagents.

Chemical Reagent Name	Purity (%)	Brand	City, Country
Glucose	AR (>99%)	Tianjin Chemical Co.	Tianjin, China
Yeast Powder	total nitrogen content ≥9.0%	Sinopharm Chemical Reagent Co., Ltd.	Shanghai, China
Potassium Dihydrogen Phosphate	AR (>99%)	Tianjin Chemical Co.	Tianjin, China
Potassium Chloride	AR (>99%)	Sinopharm Chemical Reagent Co., Ltd.	Shanghai, China
Ferrous Sulfate	AR (>99%)	Tianjin Chemical Co.	Tianjin, China
Magnesium Sulfate	AR (>99%)	Sinopharm Chemical Reagent Co., Ltd.	Shanghai, China
Citric Acid	AR (>99%)	Tianjin Chemical Co.	Tianjin, China
Sodium Nitrate	AR (>99%)	Sinopharm Chemical Reagent Co., Ltd.	Shanghai, China

**Table 2:** Instrument Parameters.

Instrument Name	Model	Manufacturer	City, Country
Electronic Balance	BSM/120.4	Shanghai Zhuojing Electronic Technology Co., Ltd.	Shanghai, China
Biochemical Incubator	ZOLY-300N	Shanghai Zhichu Instrument Co., Ltd.	Shanghai, China
Super Clean Workstation	SJ-CJ-2FD	Su Jie Medical Instrument Co., Ltd.	Suzhou, China
Pipette Controller	F1-ClipTip	Thermo Fisher Scientific	Frankfurt, Germany
High-Speed Centrifuge	JLA	Beckman Coulter, Inc.	Brea, CA, USA
pH Meter	PHS-3C	Shanghai Yi Electric Science Instrument Co., Ltd.	Shanghai, China
Electric Blast Drying Oven	DHG-9013/9053A	Shanghai Yiheng Scientific Instrument Co., Ltd.	Shanghai, China
Rotational Rheometer	Haake MARS 60	Thermo Fisher Scientific	Frankfurt, Germany
Cantilever Electric Stirrer	LC-OES-60	Shanghai Lichen Bangxi Instrument Technology Co., Ltd.	Shanghai, China

**Table 3:** Simulated brine composition.

Composition (g·L^−1^)			Total Salinity (g·L^−1^)
NaCl	CaCl_2_	MgCl_2_·6H_2_O	
192.5	16.5	11	220
70	6	4	80
5.3	0.45	0.3	6

**Table 4:** Elemental analysis of the sample.

Element	Measured Value (%)	Theoretical Value (%)
C	42.14	43.25
H	6.17	6.35
N	0.06	/
S	0.04	/
O	50.82	50.40

**Table 5:** UV absorption peak and analysis of the sample.

Solvents	Maximum Absorption Wavelength λmax (nm)	Absorbance A	Analysis
Water solution	280	0.0268	Trace protein residue

**Table 6:** Infrared absorption peak data and attribution of samples.

Absorption Peak (cm^−1^)	Intensity	Type of Vibration	Groups	Remarks
3362.46 (br)	Medium	Nu O-H	-OH	Hydroxyl
2936.82	Medium	Nu C-H	-CH2-	Alkyl (methylene)
1420.00~1200.03	Medium	Nu C-O	-C-O-	Hydroxyl
1149.13, 1079.06, 1060.86	Medium	υC-O-C, υC-O-H	C-O-C, C-O-H	Pyranose
888.48	Medium	Beta-OH	-OH	β-Configurational polysaccharide

**Table 7:** Parameters of the power law model for polymer solutions.

Polymer Concentration/ppm	k (mPa·sn)	n	R^2^	Shear Rate Range (s^−1^)
1000	94.2	0.38	0.9904	0.01 < < 1000Ý
2000	268.9	0.30	0.9925	0.01 < < 1000Ý
3000	474.7	0.26	0.9955	0.01 < < 1000Ý
4000	718.9	0.24	0.9955	0.01 < < 1000Ý
5000	1044.5	0.21	0.9982	0.01 < < 1000Ý

**Table 8:** Thermal Resistance of Common Biological Polymers [6,20].

Biological Polymer	Species	Natural Molecular Structure	Temperature Resistance
Xanthan Gum	*Xanthomonas campestris*	Double helix	10–40 °C
Dutan Gum	*Sphingomonas paucimobilis*	Double helix	100–120 °C
Scleroglucan	*Athelia rolfsii*	triple helix	Below 130 °C
Curdlan	*Alcaligenes* sp.	Double helix	25–100 °C
Gellan Gum	*Sphingomonas paucimobilis*	Parallel, double helix	20–90 °C

**Table 9:** Basic parameters of oil displacement test core.

Core Number	Length (cm)	Diameter of Section (cm)	Permeability (mD)	Pore Volume (mL)	Amount of Saturated Oil (mL)	Viscosity of Saturated Oil (cP)
JZ-13-4	29.9	3.83	105.2	70.2	41.1	2.75
JZ-13-3	29.6	3.81	116.8	71.5	41.6	2.75
JZ-13-12	30.0	3.81	118.4	70.9	41.0	2.75
JZ-13-13	29.9	3.81	110.1	70.1	40.3	2.75

**Table 10:** Experimental results of enhanced oil recovery capability of biopolymer flooding.

Core Number	Biopolymer Viscosity (mPa·s)	Water Drive Recovery (%)	Integrated Recovery Factor (%)	Enhanced Oil Recovery (%)	Resistance Factor (RF)	Residual Resistance Factor (RFF)
JZ-13-4	20	50.32	67.28	16.96	18.7	6.75
JZ-13-3	20	51.44	67.51	16.07	21.7	7.65
JZ-13-12	10	52.92	68.23	15.28	17.1	6.4
JZ-13-13	10	49.23	64.52	15.29	15.5	5.7

## Data Availability

The raw/processed data required to reproduce these findings cannot be shared at this time, as the data also forms part of an ongoing study.
